# Evaluation of Antimicrobial, Enzyme Inhibitory, Antioxidant and Cytotoxic Activities of Partially Purified Volatile Metabolites of Marine *Streptomyces* sp.S2A

**DOI:** 10.3390/microorganisms6030072

**Published:** 2018-07-18

**Authors:** Saket Siddharth, Ravishankar Rai Vittal

**Affiliations:** Department of Studies in Microbiology, University of Mysore, Manasagangotri, Mysore 570006, India; saketsiddharth@gmail.com

**Keywords:** marine actinobacteria, *Streptomyces* sp., enzyme inhibition, antimicrobial, antioxidant, cytotoxicity, GC-MS, pyrrolopyrazines

## Abstract

In the present study, marine actinobacteria *Streptomyces* sp.S2A was isolated from the Gulf of Mannar, India. Identification was carried out by 16S rRNA analysis. Bioactive metabolites were extracted by solvent extraction method. The metabolites were assayed for antagonistic activity against bacterial and fungal pathogens, inhibition of α-glucosidase and α-amylase enzymes, antioxidant activity and cytotoxic activity against various cell lines. The actinobacterial extract showed significant antagonistic activity against four gram-positive and two gram-negative pathogens. Excellent reduction in the growth of fungal pathogens was also observed. The minimum inhibitory concentration of the partially purified extract (PPE) was determined as 31.25 μg/mL against *Klebsiella pneumoniae*, 15.62 μg/mL against *Staphylococcus epidermidis*, *Staphylococcus aureus* and *Bacillus cereus*. The lowest MIC was observed against *Micrococcus luteus* as 7.8 μg/mL. MIC against fungal pathogens was determined as 62.5 μg/mL against *Bipolaris maydis* and 15.62 μg/mL against *Fusarium moniliforme*. The α-glucosidase and α-amylase inhibitory potential of the fractions were carried out by microtiter plate method. IC_50_ value of active fraction for α-glucosidase and α-amylase inhibition was found to be 21.17 μg/mL and 20.46 μg/mL respectively. The antioxidant activity of partially purified extract (PPE) (DPPH, ABTS, FRAP and Metal chelating activity) were observed and were also found to have significant cytotoxic activity against HT-29, MDA and U-87MG cell lines. The compound analysis was performed using gas chromatography-mass spectrometry (GC-MS) and resulted in three constituents; pyrrolo[1–a]pyrazine-1,4-dione,hexahydro-3-(2-methylpropyl)-, being the main component (80%). Overall, the strain possesses a wide spectrum of antimicrobial, enzyme inhibitory, antioxidant and cytotoxic activities which affords the production of significant bioactive metabolites as potential pharmacological agents.

## 1. Introduction

The microbial natural products are a source of several important drugs of high therapeutic value. Going back to the history of drugs of the first choice, it suggests that novel chemical moieties forming the backbone of bioactive compounds are primarily obtained from natural sources [1]. The microbial natural products are a source of several important drugs of high therapeutic value, namely antitumor agents [2], antibiotics [3], immunosuppressive agents [4], and enzyme inhibitors [5]. The majority of commercially available pharmaceutical products are secondary metabolites or their derivatives produced by bacteria, fungi and actinobacteria [6]. Among producers of important metabolites, actinobacteria have proven to be most prolific source accounting for more than two-third of available clinical products of several medical uses [7]. Actinobacteria are filamentous gram-positive bacteria with high G + C content [8]. They are characterized by complex morphological differentiation and are considered as an intermediate group of bacteria and fungi [9]. Their presence in various ecological habitats and marine environments has enabled research communities to exploit their tremendous potential as the richest source of pharmaceutical and biologically active products [10]. Therefore, they are contemplated as the most economical and biotechnologically beneficial prokaryotes.

Secondary metabolites are organic compounds having no direct role in the vegetative growth and the development of the organism. About 40–45% of active metabolites produced by the microorganisms are contributed by various genera of actinobacteria and are currently in clinical use [11]. Over the last few decades, actinobacterial metabolites have been used as a template for the development of anticancer agents, antibiotics, enzyme inhibitors, immunomodulators and plant growth hormones [12]. Among important genera of actinobacteria, *Streptomyces* is the most dominant and prolific source of bioactive metabolites with the broad spectrum of activity. Of 10,000 known compounds, genus *Streptomyces* alone accounts for nearly 7500 compounds, while the rare actinobacterial genera including *Nocardia*, *Micromonospora*, *Streptosporangium*, *Actinomadura*, *Saccharopolyspora* and *Actinoplanes* represent 2500 compounds [13]. Although the majority of the actinobacterial bioactive metabolites come from terrestrial habitats, recent studies on actinobacteria from diverse habitats have suggested new chemical entities and bioactive compounds [14]. Moreover, the possibility of finding a novel bioactive molecule from the terrestrial habitat has diminished over the years [15]. The marine ecosystem is an untapped and underexploited source for the discovery of novel metabolites. Species isolated from marine environments have found to be different in physiological, biochemical and molecular characteristics from their terrestrial counterparts and therefore might produce novel metabolites [16]. With the increase in resistance among pathogens and unavailability of novel metabolites from terrestrial sources, marine-derived drugs could be of great importance. However, the distribution of actinobacteria in the marine ecosystem has not been explored much and the knowledge about the marine-derived metabolites remains elusive. But, recent outbreaks about the marine actinobacterial-derived bioactive metabolites with distinct lead molecules have made a significant contribution in drug discovery and may lead to the development of new drugs in future.

The present work therefore aimed to investigate the potential of secondary metabolites produced by marine actinobacteria *Streptomyces* sp.S2A and their characterization.

## 2. Materials and Methods

### 2.1. Sample Collection

Marine sediment samples were collected from Gulf of Mannar Marine National Park (Latitude 9.127823° N, Longitude 79.466155° E), Rameshwaram, India. The collected sediment samples were brought to the laboratory in sterile zip-lock plastic bags and stored at 4 °C until further use. The sediments were pre-treated with CaCO_3_ and kept in hot air oven at 55 °C for 20 min [17].

### 2.2. Actinobacterial Isolates

Isolation of the actinobacterial strain was determined by serial dilution method on starch casein agar (Himedia, New Delhi, India) supplemented with nalidixic acid (25 μg/mL) and nystatin (50 μg/mL). The plates were incubated at 28 °C for 7 days. After incubation, individual colonies were maintained on ISP-2 slants and stored at 4 °C for further use. The ornamentation of the spore chain was analyzed by SEM.

### 2.3. Molecular Identification of Actinobacteria

Genomic DNA extraction of the strain was done using the phenol-chloroform method. The selected colony was grown in ISP-2 (International Streptomyces Project) broth on the rotary shaker (140 rpm, Hahn-Shin, Bucheon, South Korea) at 28 °C, pH 7.2 for 14 days. The cells were harvested by centrifugation at 8000 rpm for 10 min and the pellet was washed twice with normal saline. Washed pellet was suspended in 10 mM Tris-HCl (pH 8, Merck, Burlington, VT, USA) and lysozyme (2.5 mg/Ml, Sigma, Burlington, VT, USA), incubated at 37 °C for 1 h and was re-suspended in lysis buffer (50 mM Tris, 10 mM ethylenediaminetetraacetic acid (EDTA, Qualigens Fine Chemicals Pvt. Ltd., San Diego, CA, USA), 1% sodium dodecyl sulfate (SDS, Sigma)) and proteinase K (1 mg/mL, Sigma) and incubated for 1 h at 50 °C. 400 μL of phenol (Tris-saturated, Himedia, New Delhi, India) was added and mixed vigorously for 2 min. After centrifugation, the upper aqueous layer was transferred to the fresh tube (Tarsons, Kolkata, India), followed by the addition of CHCl_3_ (Sigma) and isoamyl alcohol (Merck) (24:1) and centrifuged at 1000 rpm for 15 min (4 °C). To the supernatant 50 μL of NaCl (5M) and twice the volume of absolute alcohol (Himedia) was added and kept for overnight incubation. Again, it was centrifuged at 14,000 rpm for 15 min and the pellet was washed with 70% alcohol. Pellet was air dried to remove traces of ethanol (EtOH, Himedia) and was suspended in 30 μL of Tris-EDTA (TE) buffer (Himedia). DNA was analyzed by 1% agarose gel electrophoresis (Bio-Rad, Hercules, CA, USA).

16S rRNA gene amplification was carried out using universal primer set, 27F (5′-AGAGTTTGATCCTGGCTCAG-3′) and 1492R (5′-ACGGCTACCTTGTTACGACTT-3′). The PCR conditions were programmed as follows: Initial denaturation at 95 °C for 5 min; followed by 35 cycles at 95 °C for 1 min, primer annealing at 54 °C for 1 min, extension at 72 °C for 1 min. Final extension was done at 72 °C for 10 min and was kept for cooling at 10 °C. The amplified products were determined at 1.8% agarose gel electrophoresis. The sequence was compared with similar 16S rRNA sequences obtained from BLAST search in National Center for Biotechnology Information (NCBI) database and the phylogenetic tree was constructed by the neighbor joining tree algorithm using MEGA 7.0 software (Mega, Raynham, MA, USA).

### 2.4. Fermentation

Isolate *Streptomyces* sp.S2A was inoculated in ISP-2 broth (Himedia) and kept for incubation at the rotary shaker (140 rpm, 28 °C) for 14 days. The culture broth obtained was extracted thrice with ethyl acetate (EA, Fisher Scientific, Madison, WI, USA) and concentrated under the rotary evaporator at 50 °C.

### 2.5. Antimicrobial Assays

#### 2.5.1. Disc Diffusion Method

Antimicrobial activity of active fraction was assessed by disk diffusion method against *Staphylococcus epidermidis* (MTCC 435), *Staphylococcus aureus* (MTCC 740), *Bacillus cereus* (MTCC 1272), *Escherichia coli* (MTCC 40), *Klebsiella pneumoniae* (MTCC 661), *Micrococcus luteus* (MTCC 7950) *Aspergillus flavus* (MTCC 2590), *Fusarium moniliforme* (MTCC 6576), *Bipolaris maydis* and *Alternaria alternata* (MTCC 1362). The sterile discs (6 mm, Himedia) were impregnated with 30 μL of crude extract. The pathogens were inoculated in Mueller-Hinton broth (24 h for bacteria, Himedia) and Sabouraud Dextrose both (72 h for fungi, Himedia). The well-grown bacterial and fungal cultures were plated on Mueller-Hinton agar and Potato Dextrose agar respectively (Himedia). Sterile discs loaded with extract were placed on the plate. Chloramphenicol discs (Himedia) were used as positive control for antibacterial assay, while nystatin discs (Himedia) were used for the antifungal assay. Discs impregnated with dimethyl sulfoxide (DMSO, Himedia) were used as the solvent control. The plates were incubated at 37 °C and room temperature (for test bacteria and fungi respectively) and the zone of inhibition was measured.

#### 2.5.2. Determination of Minimum Inhibitory Concentration

The minimum inhibitory concentration (MIC) value of the partially purified extract (PPE) was determined by micro dilution method. Bacterial and fungal pathogens were grown in sterile broth and 10 μL of log phase culture was added into 96 well micro titre plates. Partially purified fractions were dissolved in 1% DMSO and serially diluted to give required concentrations (1 mg/mL–3.9 μg/mL). Diluted fractions and sterile broth were added into pre-coated microbial cultures, making up a total of volume of 200 μL. The plate was incubated at 37 °C and room temperature (for test bacteria and fungi respectively).

### 2.6. Antioxidant Assays

#### 2.6.1. 2,2-diphenyl-1-picrylhydrazyl Radical Scavenging Activity (DPPH)

DPPH free radicals are highly stable and widely used to evaluate the radical scavenging activity of the antioxidants. Scavenging activity is based upon the reduction of DPPH radicals by hydrogen donating antioxidant compounds by forming DPPH-H. Radical scavenging activity of the ethyl acetate extract of the strain S2A was examined based on the previously described method by Ser et al. with minor changes [18]. Varying concentration of S2A extract was dissolved in methanol and reacted with freshly prepared DPPH solution (60 Μm, Sigma). The reaction mixture was incubated for 30 min in the dark. The absorbance was measured at 520 nm. Decreasing absorbance of DPPH solution indicates an increase in radical scavenging activity. The scavenging activity (%) was calculated using the following equation:

DPPH scavenging activity (%) = [(A_o_ − A_1_)/A_o_] × 100,

where A_o_ is the absorbance of control (blank) and A_1_ is the absorbance of the sample. Methanol was used as a blank whereas trolox (Sigma) was used as the reference compound [19].

#### 2.6.2. Metal Chelating Activity

The metal chelating activity was examined by measuring the ability of the compound to compete with ferrozine for Fe^2+^, complex of which can be quantified spectrophotometrically. Metal chelating activity was measured by the method previously described by Adjimani and Asare with minor modifications [20]. Assay measures the reduction in the color intensity as a result of disruption of ferrous ion and ferrozine complexes. Briefly, varying concentration of extract was added to 0.15 mL of 2 mM FeCl_2_. The reaction was initiated with the addition of 5 mM ferrozine (Sigma), followed by the incubation at the room temperature for 10 min. The absorbance was measured at 562 nm. The percentage of inhibition was calculated using the following equation:

Metal chelating activity (%) = [(A_o_ − A_1_)/A_o_] × 100,

where A_o_ is the absorbance of control and A_1_ is the absorbance of the sample. EDTA was used as a positive control.

#### 2.6.3. 2,2′-Azino-bis(3-ethylbenzothiazoline-6-sulfonic acid) Radical Scavenging Activity (ABTS)

ABTS (Sigma) scavenging activity is based upon the reduction of ABTS* radicals by compounds having lower redox potential than that of ABTS. The 2,2′-azino-bis(3-ethylbenzothiazoline-6-sulfonic acid) (ABTS) radical scavenging assay was carried out according to the method developed by Ser et al. [21]. Initially, ABTS stock solution (7 mM) was mixed with potassium persulfate (2.45 Mm, Himedia) to form ABTS cation complex for 12 h. The ABTS complex solution was added to varying concentrations of the extract preloaded in a 96-well microplate. The reaction was kept for incubation at room temperature for 20 min and the absorbance was measured at 734 nm. The percentage scavenging activity was calculated using the following formula:

ABTS radical scavenging activity (%) = [(A_o_ − A_1_)/A_o_] × 100,

where A_o_ is the absorbance of control and A_1_ is the absorbance of the sample. Trolox (Sigma) was used as a positive control.

#### 2.6.4. Ferric Reducing Antioxidant Power (FRAP) Assay

This assay determined the reduction of ferric ions to ferrous ions which was monitored spectrophotometrically at 593 nm. The FRAP assay was performed according to the method previously described by Benzy and Strain with minor modification [22], based on the reduction of ferric complex to ferrous complex by the antioxidants. Initially, FRAP reagent was prepared by adding acetate buffer (pH 3.6), 10 mM TPTZ (Sigma) and 20 mM FeCl_3_ (Himedia) at a ratio of 10:1:1. The reaction was started with the addition of varying concentration of extracts to the FRAP reagent. The mixture was then incubated at 37 °C for 10 min and absorbance was measured at 593 nm. Trolox was used as the positive control. The final FRAP values were expressed as Trolox equivalent antioxidant capacity (μM TE/g sample).

### 2.7. Enzyme Inhibitory Activities

#### 2.7.1. Inhibition Assays for α-glucosidase Activity

The α-glucosidase inhibition was determined by the 96-well microtiter plate method based on the calorimetric assay as previously described by Vinholes et al. [23]. α-glucosidase enzyme solution (2U mL^−1^, Sigma) was prepared in 100 mM phosphate buffer (pH 7.0). Ethyl acetate extracts were used in concentrations ranging from 10–100 μg mL^−1^. 2 mM of *para*-nitrophenyl-α-d-glucopyranoside (Sigma) was prepared in 50 mM phosphate buffer (pH 7.0). 50 μL of the partially purified fraction was pre-incubated with an equal volume of yeast enzyme at 37 °C for 5 min, followed by the addition of 30 μL of pNPG and further incubation for 30 min. After incubation, 100 μL of stopping reagent (0.1 M Na_2_CO_3_) was added to cease the reaction. Color produced was quantified by UV spectrophotometer (Shimadzu, Kyoto, Japan) at 405 nm. Each experiment was performed in triplicate. Acarbose (Sigma) was used as a positive control, whereas purified fraction was replaced by phosphate buffer in control. Reaction mixture without enzyme was taken as blank. The percentage inhibition (%) was determined by the formula:

$$\mathrm{percentage inhibition}\;(\%)=\frac{\mathrm{absorbance of control}-\mathrm{absorbance of sample}}{\mathrm{absorbance of control}}\times 100$$

#### 2.7.2. Inhibition Assays for α-amylase Activity

The α-amylase inhibition was determined by 96-well microtiter plate method based on calorimetric assay as previously described by Balasubramaniam et al. [24]. Equal volume of test samples (5 mg mL^−1^) and α-amylase solution (0.5 mg mL^−1^, Sigma) prepared in 30 mM phosphate buffer (pH 7.0) was pre- incubated at 37 °C for 10 min. 50 μL of 0.5% starch solution was added and incubated for 10 min at 37 °C. 120 μL of DNS reagent (Sigma) was added to stop the reaction. The reaction mixture was incubated at 95 °C for 5 min, cooled to room temperature. Absorbance was measured at 540 nm in a microplate reader. Acarbose at the concentration 2 mg mL^−1^ was taken as positive control. The inhibition percentage of amylase was determined by the formula reported in the previous paragraph.

### 2.8. Cytotoxicity Assay

The human cell lines HT-29 (Colon cancer), MDA (Breast cancer) and U-87 MG (Brain cancer) were procured from National Centre for Cell Science, Pune, India. The cell lines were cultured and maintained in Dulbecco’s modified Eagle’s medium (DMEM, Sigma) in T-flasks in the incubator at 37 °C and internal atmosphere of 95% air and 5% CO_2_. The cytotoxicity was determined by standard MTT dye assay, according to the method described by Carmichael et al. [25]. Briefly, varying concentration of extracts were dissolved in 1% DMSO and treated to cells seeded in 96 well tissue culture plates. The plates were kept for incubation at 37 °C for 24 h, MTT solution (Sigma) was added and incubated for 4 h at 37 °C. The amount of purple formazan crystals resulting from the reduction of MTT dye by succinic dehydrogenase in mitochondria of the viable cells was determined by measuring OD at 570 nm. The IC_50_ value was calculated using graph pad prism. Each assay was performed in triplicate.

### 2.9. Gas Chromatography-Mass Spectrometry (GC-MS)

The analysis of the volatile constituents in extracts was determined by GC-MS technique (Perkin Elmer Clarus, USA). Perkin Elmer Clarus 680 employed a fused silica column, packed with Elite-5MS and the compounds were separated using helium as a carrier gas at a constant flow of 1 mL/min. The injector temperature was kept at 260 °C. Oven temperature was set as follows: 60 °C (2 min); followed by 300 °C at the rate of 10 °C min^−1^. The spectrum thus obtained was compared with the database of the already known spectrum of components stored in GC-MS NIST library. The infrared spectrum of the extract was analyzed by FT-IR spectrophotometer in the range of 400–4000 cm^−1^.

## 3. Results

### 3.1. Isolation and Molecular Identification of the Strain

**Figure 1:**
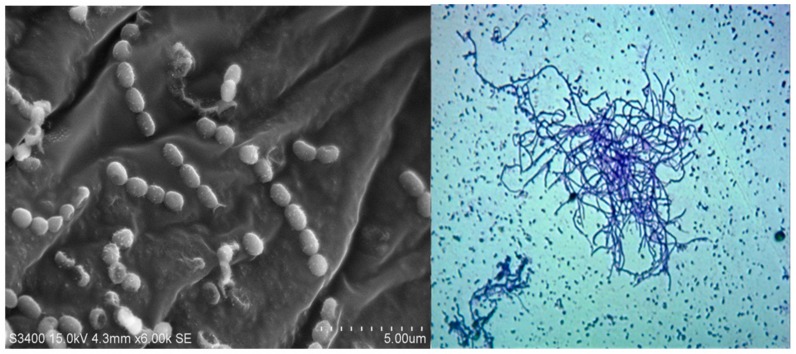
(**A**) Scanning electron micrograph showing spore ornamentation in *Streptomyces* sp.S2A; (**B**) Microscopic image of *Streptomyces* sp.S2A under 100×.

**Figure 2:**
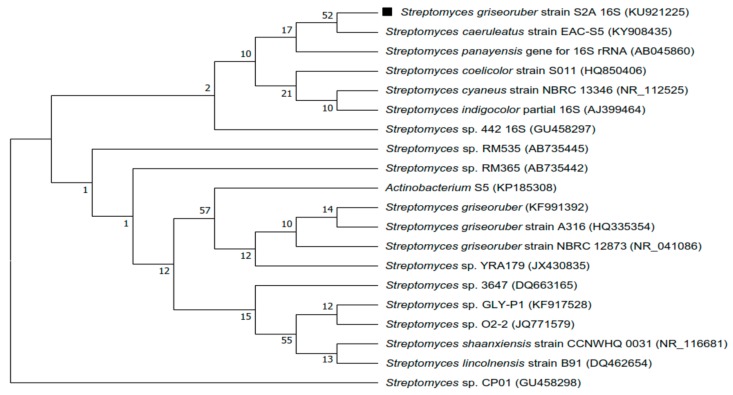
Phylogenetic tree of *Streptomyces* sp.S2A and the relationships with the closest species based on 16S rRNA gene sequencing using the neighbor-joining method.

Marine sediment from Gulf of Mannar was pre-treated with physical and chemical methods. Grown on SCA and ISP-2 medium, the cultural characteristics were identical on either of them. The aerial hyphae were white in color and substrate mycelium was colorless. SCA and ISP-2 culture plates did not show any pigment diffusion. Micromorphological studies of strain using SEM showed smooth spore ornamentation and rectiflexibilis spore morphology (Figure 1). The genomic DNA of the strain was isolated using the phenol-chloroform method and examined for 16S r-RNA sequence. The amplified sequences were subjected to BLAST analysis using the megablast tool of Genebank at NCBI under the accession number (KU921225). The BLAST search revealed that the strain belonged to *Streptomyces* sp. The highest similarity value index was found between the sequences of *Streptomyces* sp.S2A and *Streptomyces griesoruber* (100%). The neighbor-joining phylogenetic tree was drawn using MEGA 7.0 (Figure 2).

### 3.2. Antimicrobial Assays

#### 3.2.1. Disc Diffusion Method

Antagonistic characteristics of the bioactive extract of *Streptomyces* sp.S2A showed potent antagonistic activity against bacterial and fungal pathogens (Table 1). Of six bacterial pathogens, the highest inhibition activity was manifested against *Micrococcus luteus* and *Staphylococcus epidermidis* (16 mm). Susceptibility of *Bacillus cereus*, *Klebsiella pneumoniae* and *Staphylococcus aureus* to bioactive compounds was highly noticeable (14 mm). *Escherichia coli* was less susceptible to the compound (10 mm). Among fungal pathogens, reduction in mycelial growth was not seen against *Aspergillus flavus* and *Alternaria alternata* whereas inhibitory activity was significantly observed against *Fusarium moniliforme* and *Bipolaris maydis* (See Supplementary Figure S1).

#### 3.2.2. Determination of Minimum Inhibitory Concentration (MIC)

The minimum inhibitory concentration of the extract was determined as 31.25 μg/mL against *Klebsiella pneumoniae*, 15.62 μg/mL against *Staphylococcus epidermidis*, *Staphylococcus aureus*, *Bacillus cereus* and *Escherichia coli*. Lowest MIC was observed against *Micrococcus luteus* as 7.8 μg/mL. The solvent DMSO (1%) had no significant inhibitory activity against pathogens. MIC against fungal pathogens was determined as 62.5 μg/mL against *Bipolaris maydis* and 15.62 μg/mL against *Fusarium moniliforme*. (Table 1).

### 3.3. Antioxidant Assays

#### 3.3.1. DPPH Radical Scavenging Activity

The highest inhibition concentration of radical scavenging activity of the extract was found to be 56.55 ± 3.1%, as compared to the trolox that was found to be 74.73 ± 1.13%. The IC_50_ value for DPPH radical scavenging activity of the extract was 0.86 mg (Table 2).

#### 3.3.2. Metal Chelating Activity

The study showed the decrease in the formation of ferrozine-Fe^2+^ complex with increase in the concentration of the extract. It showed the significant chelating activity measuring from 18.40 ± 1.4% to 59.98 ± 2.12% at the concentration ranging from 0.25–2 mg/mL (Table 2).

#### 3.3.3. ABTS Radical Scavenging Activity

This assay showed the significant increase in the scavenging activity with increase in the concentration of the extract, thus decolorized the blue-green color of ABTS* back into ABTS, which is colorless. The IC_50_ value for ABTS radical scavenging activity of the extract was 11.77 μg (Table 2).

#### 3.3.4. Ferric Reducing Antioxidant Power (FRAP) Assay

The increase in absorbance was observed with the increase in the concentration of the extract suggesting the significant antioxidant activity (Table 2).

### 3.4. In Vitro Enzyme Inhibition Assay

The EA extract exhibited α-amylase and α-glucosidase inhibitory activity in a dose-dependent manner. Acarbose was used as a standard. IC_50_ value of EA extract for α-glucosidase and α-amylase inhibition was found to be 21.17 and 20.46 respectively, whereas that of acarbose was 15.47 and 18.15 μg/mL respectively (Table 3 and Table 4).

### 3.5. Cytotoxicity Assay

The tested results of the extract against cell lines were shown in (See Supplementary Figure S2). The results revealed that the extract showed varying efficacy against cell lines. The highest activity against U-87 at 100 μg/mL was found to be 59.63 ± 1.9%. It also showed significant activity against MDA and HT-29 with cell inhibition was found to be 55.23 ± 1.09% and 52.31 ± 2.4% respectively at 100 μg/mL (Table 5). Overall, the results suggested the potential cytotoxic activity against various cell lines.

### 3.6. Gas Chromatography-Mass Spectrometry (GC-MS)

**Figure 3:**
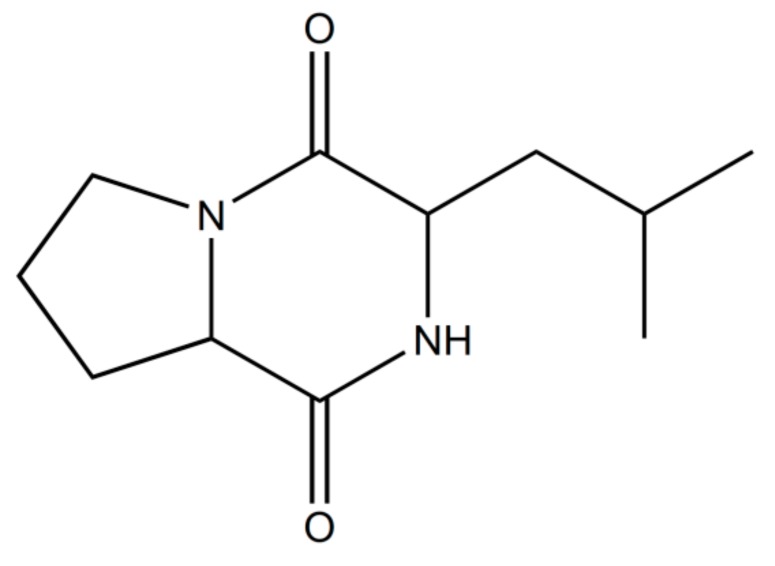
Chemical structure of the compound pyrrolo[1–a]pyrazine-1,4-dione,hexahydro-3-(2-methylpropyl).

**Figure 4:**
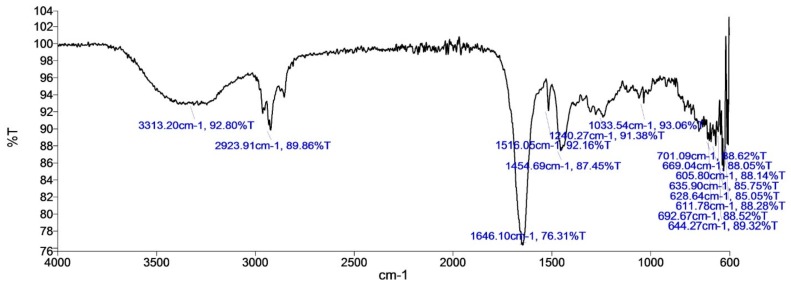
FT-IR spectrum of the active extract of *Streptomyces* sp.S2A.

Analysis of components of the active fraction with the highest activity by GC-MS analysis implied nine peaks at the retention time of (i) 17.239; (ii) 17.309; (iii) 20.811; (iv) 21.311; (v) 21.406; (vi) 21.586; (vii) 22.071; (viii) 22.126; (ix) 24.257. Further examination of MS peaks revealed *m*/*z* at 168, 259, 210 and 350. According to NIST library search, peak retentions at 21.311, 21.406 and 21.586 correspond to single compound i.e., pyrrolo[1–a]pyrazine-1,4-dione,hexahydro-3-(2-methylpropyl) (Figure 3). Other tentatively identified compounds were diphenylmethane, 2-Isopropyl-1-Phenyl-3-Pyrrolidin-1-yl Propane-1,3-Dione and Benzene, 1′1-tetradecyclidenebis (The GC-MS spectrum indicated the ions at 70 and 154 corresponded to molecule C_7_H_10_NO_2_ and C_4_H_8_ ions (See Supplementary Figures S3 and S4). The spectrum was similar to that of pyrrolo[1–a]pyrazine-1,4-dione,hexahydro-3-(2-methylpropyl) spectra in the GC-MS library and a study reported by Yang et al. [26]. The FT-IR spectrum of the partially purified metabolite showed the characteristic functional groups such as NH stretching peak of a primary amine at 3313.20 cm^−1^. The functional group at 2923.91 cm^−1^ corresponded to strong C-H stretching in alkanes. The peak at 1646.10 cm^−1^ was assigned to C-N stretch in primary amine. The absorption peak at 1516.05 cm^−1^ was assigned to C=C stretch in an alkene. The peak at 1454.69 cm^−1^ was assigned to C-H bend in alkanes. The peak at 1240.27 cm^−1^ was assigned to C-O stretch. The absorption peak at 1033.54 cm^−1^ was assigned to the ether. The peak ranging from 605.8 cm^−1^ to 701.09 cm^−1^ was assigned to strong C-H bend in alkenes (Figure 4).

## 4. Discussion

With this outlook, the present investigation was carried out to identify bioactive compound from marine actinobacteria exhibiting antagonistic activity against bacterial and fungal pathogens, enzyme inhibition activity, antioxidant and cytotoxic activity. *Streptomyces* sp.S2A isolated from marine sediment of Gulf of Mannar produced white aerial mycelium to colorless substrate mycelium on SCA medium. Shirling and Gottileb [27] reported that the pigmentation paradigm could be used for the classification and identification. However, there was no pigment pattern observed with the isolate. Extraction of metabolites with ethyl acetate yielded the dark color residue. Purification by silica gel column chromatography resulted in six fractions, of which one fraction exhibited significant activity. The antagonistic activity of the bioactive compound showed the high zone of inhibition against *Micrococcus luteus* and *Staphylococcus epidermidis* (16 mm). Moderate activity was observed against *Bacillus cereus*, *Klebsiella pneumoniae* and *Staphylococcus aureus* (14 mm). Susceptibility of *Escherichia coli* to the compound was found to be weaker (10 mm). Inhibitory potential of the bioactive compound against fungal pathogens showed good activity against *Fusarium moniliforme* and *Bipolaris maydis*, whereas no zone of inhibition was observed against *Aspergillus flavus* and *Alternaria* *aleternata*. MIC was used to determine the efficacy of the compound at different concentration. The reduction in the growth of bacterial pathogens was observed with the concentration ranging from 7.8–31.25 μg/mL. MIC of the compound showed excellent antifungal activity against *Fusarium moniliforme* and *Bipolaris maydis*. Ethyl acetate extract of *Streptomyces* sp.S2A also showed significant α-glucosidase and α-amylase inhibition activity, though IC_50_ of the extract were less than that of acarbose. Antioxidant and cytotoxic activities of the extract was determined by the DPPH, ABTS, FRAP and metal chelating assays and against HT-29, MDA and U-87 MG cell lines. The results thus obtained showed the presence of potent antioxidant and anticancer agents.

The partial chemical composition relates to the metabolite was detected by GC-MS. The chromatogram of fraction A43 showed a total of nine peaks. Of all, the major constituent was pyrrolo[1–a]pyrazine-1,4-dione,hexahydro-3-(2-methylpropyl)-, constituted 80.7% and this may be the active principle compound. The other chemical compound was identified as diphenylmethane (6%), 2-Isopropyl-1-Phenyl-3-Pyrrolidin-1-yl-Propane-1,3-Dione (2%) and Benzene, 1′1-tetradecyclidenebis (2%). Pyrrolo[1–a]pyrazine-1,4-dione,hexahydro-3-(2-methylpropyl is a peptide derivative of diketopiperazine with the molecular weight as 210 and empirical formula as C_11_H_18_N_2_O_2_. All the bioassays mentioned in the above paragraph were confirmed with the commercially available purified compound: pyrrolo[1–a]pyrazine-1,4-dione,hexahydro-3-(2-methylpropyl). Antimicrobial and the cytotoxic activity of the purified compound were found to be significantly higher the partially purified compound, whereas the enzyme inhibition potential of the partially purified compound against α-glucosidase and α-amylase were better in comparison with commercial compound. The mass spectrum of the commercial compound corresponds to 70.0315 *m*/*z* and 154.0152, which is same as shown by the compound present in partially purified extract (See Supplementary Figures S5 and S6, Tables S1–S3).

Pyrrolopyrazines are known for their wide range of biological activities such as antioxidant, anti-angiogenesis, anti-tumor and antimicrobial [28]. Manimaram et al. reported the presence of antibacterial metabolite, pyrrolo[1–a]pyrazine-1,4-dione,hexahydro-3-(2-methylpropyl) in the crude extract of *Streptomyces* sp. VITMK1 isolated from mangrove soil [29]. Antifouling potential of pyrrolo[1–a]pyrazine-1,4-dione,hexahydro-3-(2-methylpropyl) against *Vibrio halioticoli* and *Loktanella honkongensis* was studied by Dash et al. [30]. Sponge-derived marine bacteria significantly inhibited the larval settlement of *Balanus amphitrite* and *Hydroides elegans*. Another marine bacteria isolated from the sponge, *Spongia officinalis* showed the potent antibacterial and antifungal activity of pyrrolo[1–a]pyrazine-1,4-dione,hexahydro-3-(2-methylpropyl) [31]. Diketopiperzines derivatives present in marine *Streptomyces* sp. had shown good anti-H1N1 activity [32]. Mithun and Rao also reported the presence of pyrrolopyrazines in *Micrococcus luteus* with anti-cancer activity against HCT-15 cell line [33]. Presence of pyrrolo[1–a]pyrazine-1,4-dione,hexahydro-3-(2-methylpropyl) was detected in *Streptomyces* sp. MUM 256 isolated from the mangrove forest in Malaysia. This compound was reported to possess antioxidant and anticancer activities [34]. Anti-cancer metabolites were also reported from *Streptomyces malaysiense* sp.MUSC 136 isolated from the mangrove ecosystem. The bioactive metabolite exhibited strong antioxidant activity and high cytotoxic activity against HCT-116 cells [35]. The first report on marine *Staphylococcus* sp. derived pyrrolo[1–a]pyrazine-1,4-dione,hexahydro-3-(2-methylpropyl) was reported by Lalitha et al. [36]. Purified metabolite was potentially active against lung (A549) and cervical (HeLa) cancer cells in a dose-dependent manner. Thus, the present study suggested that the pyrrolopyrazines derivative pyrrolo[1–a]pyrazine-1,4-dione,hexahydro-3-(2-methylpropyl) may account for the observed antagonistic, antioxidant, and cytotoxic activities in marine actinobacteria, *Streptomyces* sp.S2A. The results obtained in the current study demonstrate that bioactive metabolites produced by marine actinobacteria have tremendous potential for pharmaceutical product and are a subject of future investigation.

## Figures and Tables

**Table 1:** Antimicrobial activity and MIC (μg/mL) of *Streptomyces* sp.S2A by broth dilution method.

Test Microorganisms	Zone of Inhibition (mm)		MIC (μg/mL)
**Bacteria**	**Extract**	**Antibiotics (Chloramphenicol)**	
*Klebsiella pneumoniae* MTCC 661	14 ± 0.4	30 ± 1.1	31.25
*Micrococcus luteus* MTCC 7950	16 ± 0.8	28 ± 1.6	7.81
*Escherichia coli MTCC* 40	10 ± 0.8	22 ± 1.9	15.62
*Bacillus cereus* MTCC 1272	14 ± 1.2	25 ± 1.1	15.62
*Staphylococcus epidermidis* MTCC 435	16 ± 0.4	23 ± 1.8	15.62
*Staphylococcus aureus* MTCC 740	14 ± 0.8	24 ± 0.8	15.62
**Fungi**		**(Nystatin)**	
*Aspergillus flavus* MTCC 2590	-	-	-
*Bipolaris maydis*	14 ± 1.2	20±1.2	31.25
*Alternaria alternata* MTCC 1362	-	-	-
*Fusarium moniliforme* MTCC 6576	18 ± 1.2	22±1.0	7.81

**Table 2:** Radical scavenging activity of ethyl acetate extract of *Streptomyces* sp.S2A.

Antioxidant Assays	Concentration of Extract (mg/mL)	% Inhibition	Absorbance	IC_50_ (mg/mL)
DPPH	1.0	56.55 ± 3.1	-	
	0.50	32.33 ± 1.4	-	0.86
	0.25	17.29 ± 1.6	-	
Metal chelating	2.0	59.98 ± 2.12	-	
	1.0	37.50 ± 2.36	-	1.56
	0.50	24.90 ± 2.11	-	
	0.25	18.40 ± 1.4	-	
ABTS	0.10	42.48 ± 3.1	-	
	0.05	30.24 ± 3.74	-	0.011
	0.02	7.29 ± 3.62	-	
FRAP	0.1	-	0.248	
	0.08	-	0.202	
	0.06	-	0.145	-
	0.04	-	0.060	
	0.02	-	0.028	

**Table 3:** α-glucosidase inhibition and IC_50_ values of ethyl acetate extract of *Streptomyces* sp.S2A.

Concentration (μg/mL)	Inhibition % (EA Extract)	IC_50_ (μg/mL) (EA Extract)	Inhibition % (Acarbose)	IC_50_ (μg/mL) (Acarbose)
6.25	29.12 ± 0.33		36.44 ± 0.58	
12.5	38.54 ± 0.77		45.27 ± 0.34	
25	55.1 ± 1.16	21.17	62.19 ± 1.10	15.47
50	68.4 ± 1.55		78.52 ± 1.99	
100	72.31 ± 1.01		86.83 ± 2.01	
200	81.74 ± 2.65		94.22 ± 2.33	

**Table 4:** α-amylase inhibition and IC_50_ values of ethyl acetate extract of *Streptomyces* sp.S2A.

Concentration (μg/mL)	Inhibition % (EA Extract)	IC_50_ (μg/mL) (EA Extract)	Inhibition % (Acarbose)	IC_50_ (μg/mL) (Acarbose)
6.25	16.44 ± 0.21		20.19 ± 0.78	
12.5	34.77 ± 0.44		40.05 ± 0.10	
25	59.29 ± 1.15	20.46	64.44 ± 1.45	18.15
50	74.32 ± 1.09		87.57 ± 1.33	
100	81.13 ± 1.34		97.03 ± 1.10	
200	88.67 ± 1.93		97.84 ± 1.78	

**Table 5:** Cytotoxic activity of extract of *Streptomyces* sp.S2A against HT-29, MDA and U-87 MG.

Concentration (μg/mL)	Inhibition %		
	U-87 MG	MDA	HT-29
5	13.76 ± 1.81	3.57 ± 1.76	18.51 ± 3.89
10	16.51 ± 2.01	10.71 ± 3.75	21.76 ± 2.32
20	19.26 ± 3.79	15.0 ± 4.10	23.15 ± 1.96
50	36.19 ± 2.11	30.95 ± 2.87	35.31 ± 2.77
100	59.63 ± 1.90	55.23 ± 1.09	52.31 ± 2.40
**IC_50_ (μg/mL)**	93.32	80.02	88.68

## Supplementary Materials

The following are available online at http://www.mdpi.com/2076-2607/6/3/72/s1.
