# Supplementary material for: Evaluation of Antimicrobial, Enzyme Inhibitory, Antioxidant and Cytotoxic Activities of Partially Purified Volatile Metabolites of Marine Streptomyces sp.S2A
Source: Microorganisms. 2018 Jul 18;6(3):72. doi: 10.3390/microorganisms6030072 (PMC6163298; doi:10.3390/microorganisms6030072)
Supplement: Supplementary file 1 [file microorganisms-06-00072-s001.pdf]

<u>**Supplementary Information**</u>

Evaluation of Antimicrobial, Enzyme Inhibitory, Antioxidant and Cytotoxic Activities of Partially Purified Volatile Metabolites of Marine *Streptomyces* sp.S2A

Saket Siddharth and Ravishankar Rai V*
Department of studies in Microbiology, University of Mysore, Manasagangotri, Mysore – 570006, INDIA

*Correspondence: Ravishankar Rai V, Department of Studies in Microbiology, University of Mysore, Manasagangotri, Mysore - 570 006, India. Tel: +91821-2419441; E-mail: saketsiddharth@gmail.com; raivittal@gmail.com

Contents:

Figure S1. Antimicrobial activity of the bioactive extract of the *Streptomyces* sp.S2A on pathogens. (A) *Escherichia coli* (B) *Klebsiella pneumoniae* C) *Micrococcus luteus* (D) *Bacillus cereus* (E) *Staphylococcus aureus* (F) *Staphylococcus epidermidis* (G) *Bipolaris maydis* (H) *Fusarium moniliforme.*

Figure S2. Cytotoxic effect of extract against (A) HT-29 (B) MDA (C) U-87 MG. Morphological studies show that after treatment with extract, cells shrunk with the formation of crystals and detached from the surface.

Figure S3. GC-Mass spectrum of pyrrolo[1,2-a]pyrazine-1,4-dione,hexahydro-3-(2-methylpropyl).

Figure S4. Gas chromatographic spectra of the active extract of *Streptomyces* sp.S2A.

Figure S5. Cytotoxic effect of pyrrolo[1,2-a]pyrazine-1,4-dione,hexahydro-3-(2-methylpropyl) (Commercial compound) against (A) HT-29 (B) MDA (C) U-87 MG. Morphological studies show that after treatment with compound, cells shrunk with the formation of crystals and detached from the surface significantly.

Figure S6. Mass spectrum of pyrrolo[1,2-a]pyrazine-1,4-dione,hexahydro-3-(2-methylpropyl) (Commercial compound).

Table S1. Antimicrobial activity and MIC (μg/mL) of pyrrolo[1,2-a]pyrazine-1,4-dione,hexahydro-3-(2-methylpropyl) (Commercial compound) by broth dilution method.

Table S2. α-glucosidase inhibition and IC50 values of pyrrolo[1,2-a]pyrazine-1,4-dione,hexahydro-3-(2-methylpropyl) (Commercial compound)**.**

Table S3. α-amylase inhibition and IC50 values of pyrrolo[1,2-a]pyrazine-1,4-dione,hexahydro-3-(2-methylpropyl) (Commercial compound).

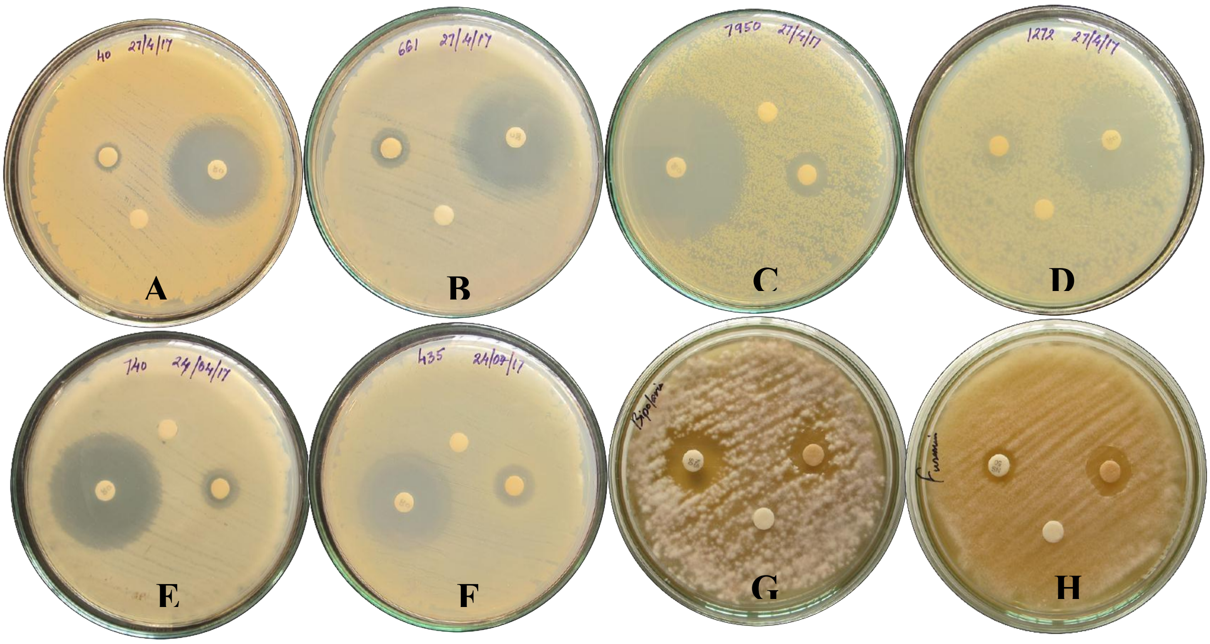

Figure S1. Antimicrobial activity of the bioactive extract of the *Streptomyces* sp. on pathogens. **(A)** *Escherichia coli* **(B)** *Klebsiella pneumoniae* **C)** *Micrococcus luteus* **(D)** *Bacillus cereus* **(E)** *Staphylococcus aureus* **(F)** *Staphylococcus epidermidis* **(G)** *Bipolaris maydis* **(H)** *Fusarium moniliforme*.

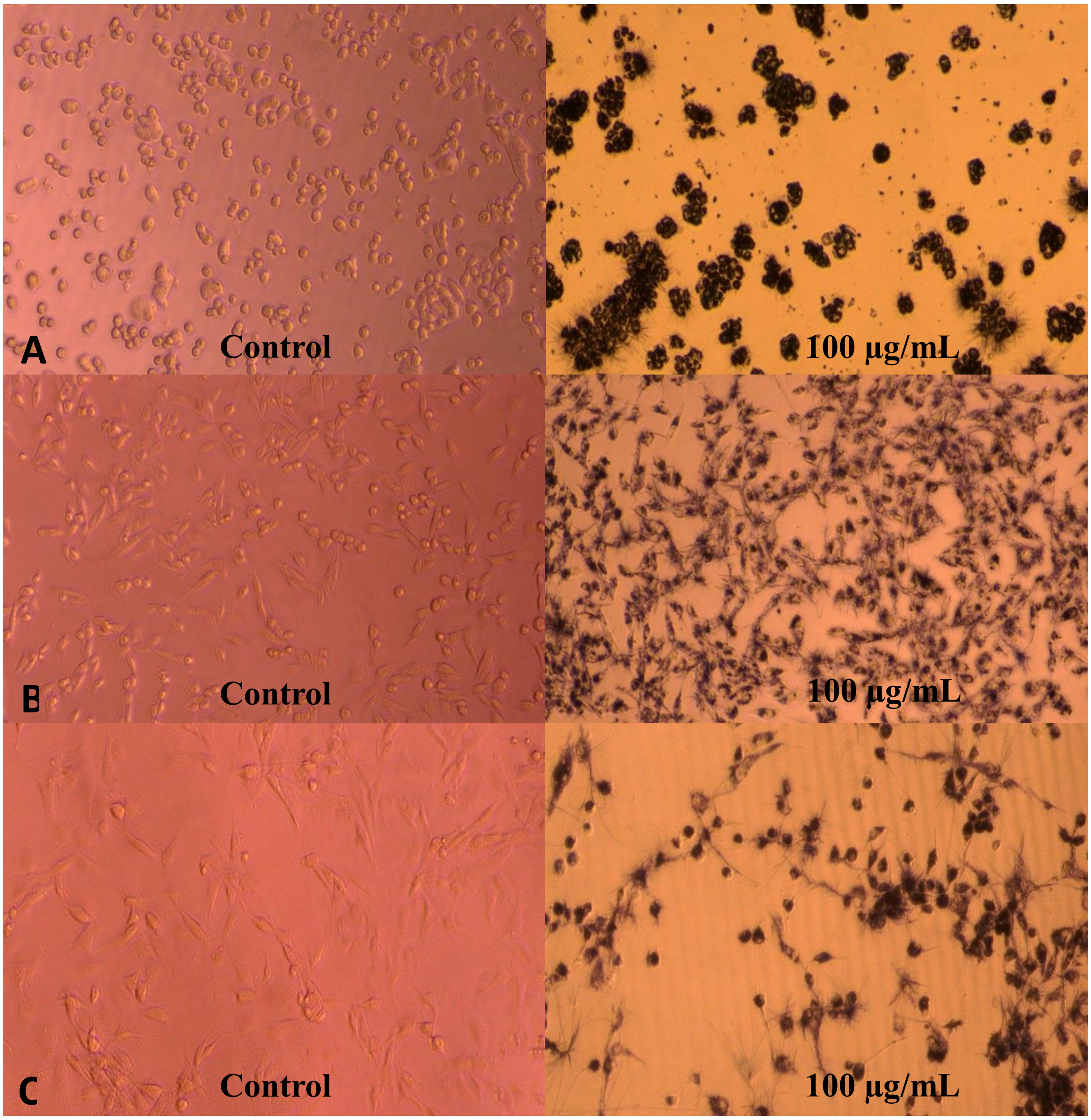

Figure S2. Cytotoxic effect of extract against **(A)** HT-29 **(B)** MDA **(C)** U-87 MG. Morphological studies show that after treatment with extract, cells shrunk with the formation of crystals and detached from the surface.

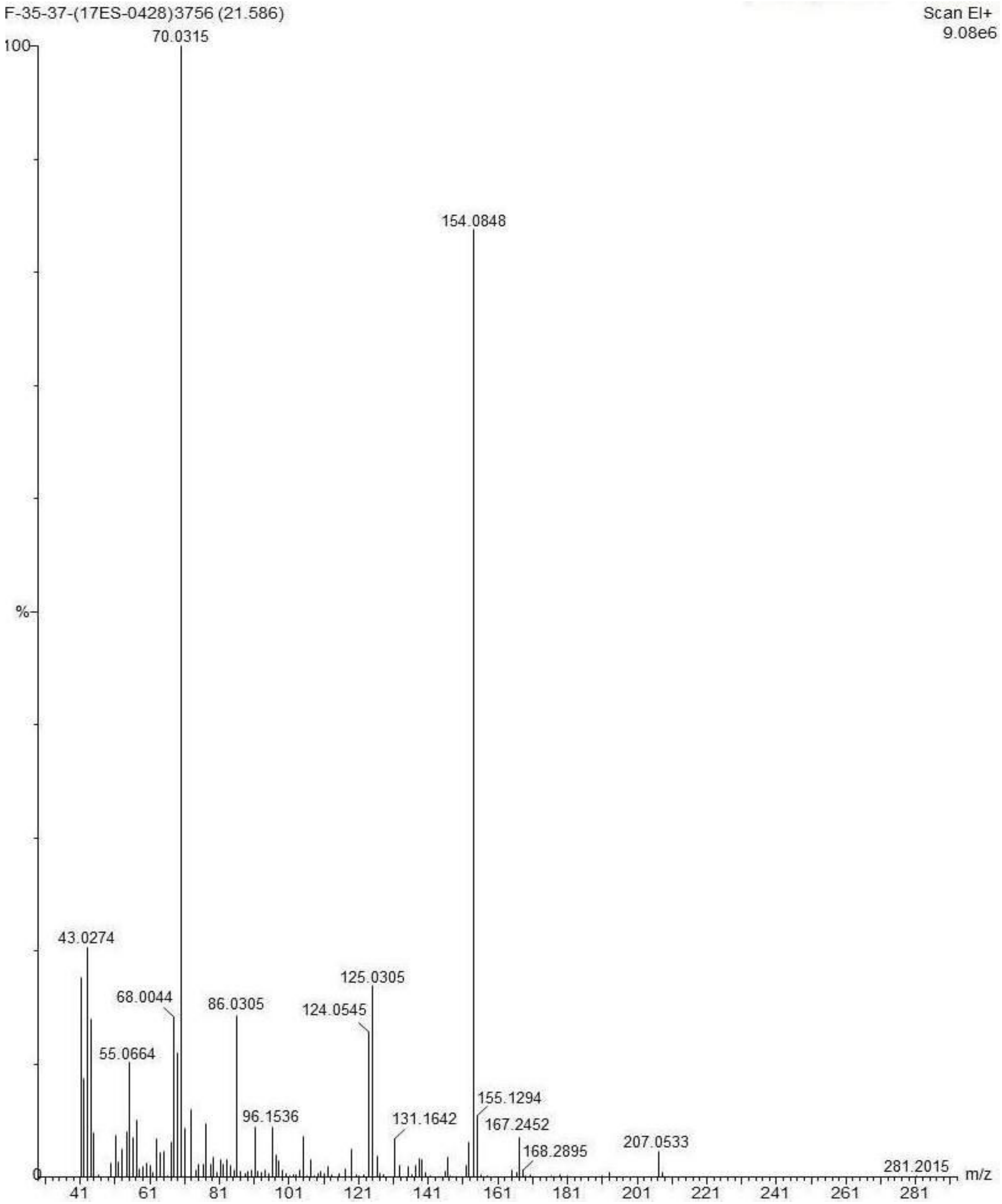

Figure S3. GC-Mass spectrum of pyrrolo[1,2-a]pyrazine-1,4-dione,hexahydro-3-(2-methylpropyl).

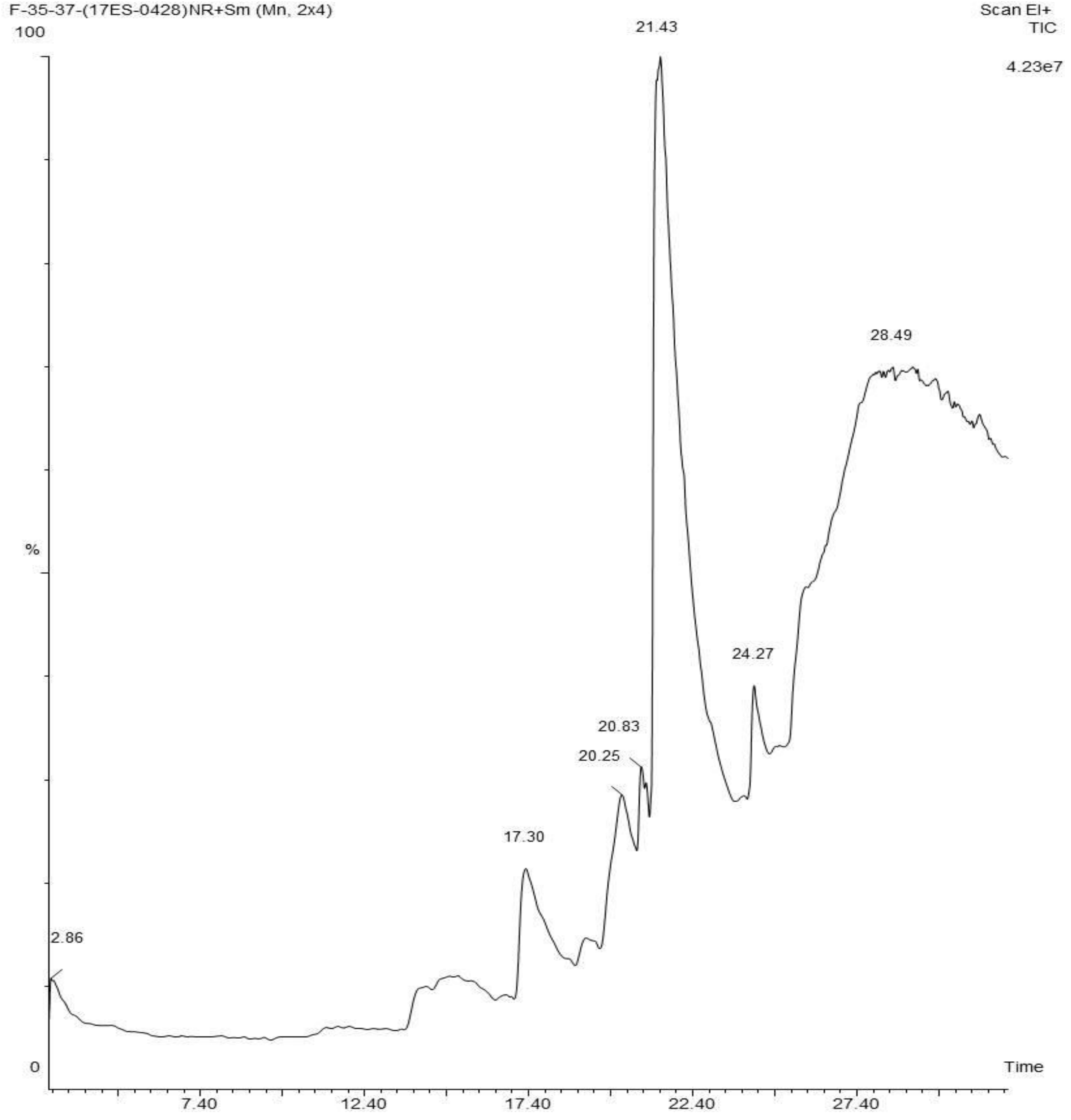

Figure S4. Gas chromatographic spectra of the active extract of *Streptomyces* sp.S2A.

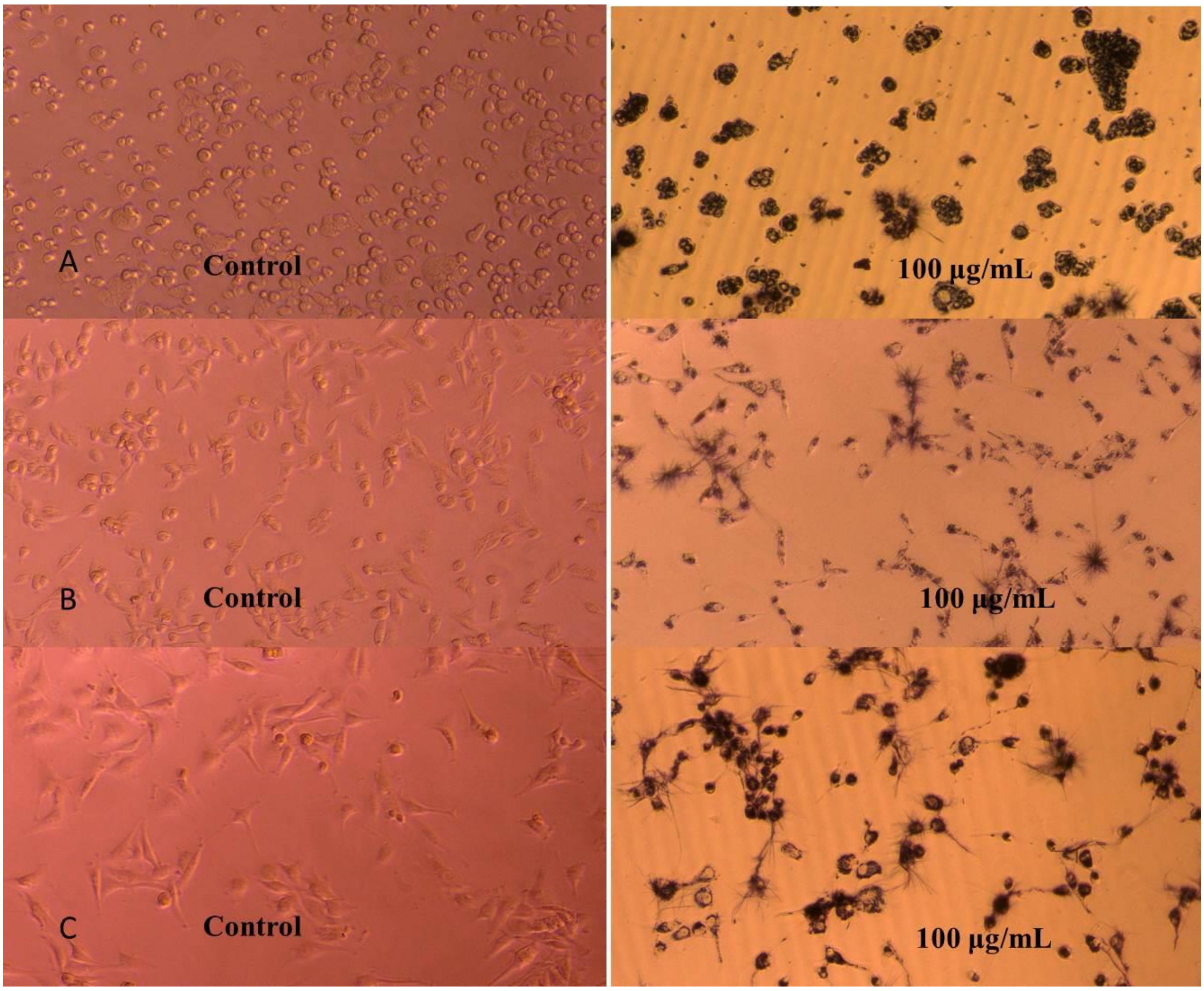

Figure S5. Cytotoxic effect of pyrrolo[1,2-a]pyrazine-1,4-dione,hexahydro-3-(2-methylpropyl) (Commercial compound) against **(A)** HT-29 **(B)** MDA **(C)** U-87 MG. Morphological studies show that after treatment with compound, cells shrunk with the formation of crystals and detached from the surface significantly.

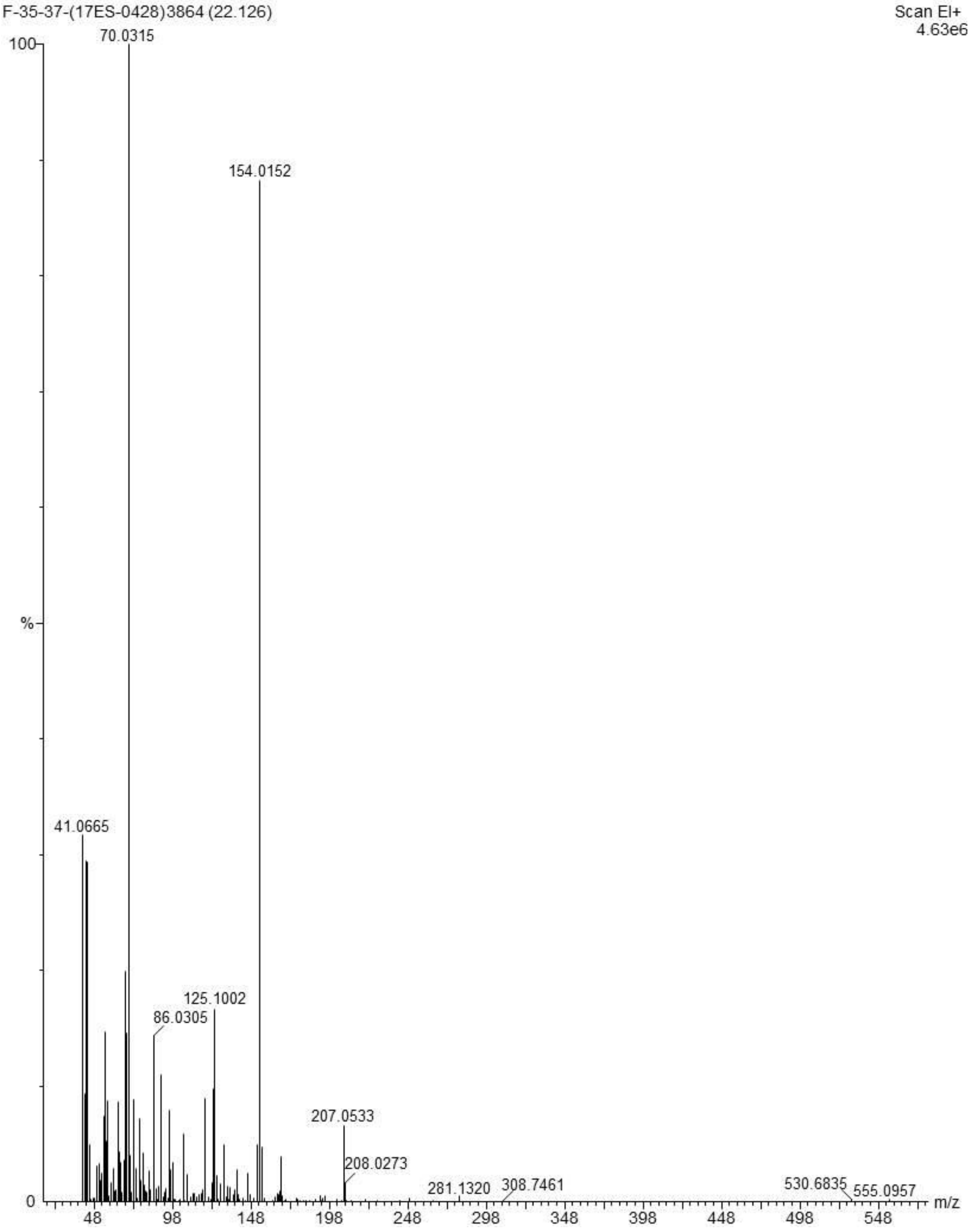

Figure S6. Mass spectrum of pyrrolo[1,2-a]pyrazine-1,4-dione,hexahydro-3-(2-methylpropyl) (Commercial compound)

Table S1. Antimicrobial activity and MIC (µg/mL) of pyrrolo[1,2-a]pyrazine-1,4-dione,hexahydro-3-(2-methylpropyl) (Commercial compound)by broth dilution method.

Test microorganisms	Zone of inhibition (mm)		MIC (µg/mL)
**Bacteria**	**Commercial compound**	**Antibiotics (Chloramphenicol)**	
Klebsiella pneumoniae MTCC 661	20±1.2	28±0.6	7.81
Micrococcus luteus MTCC 7950	19±0.2	24±0.4	1.95
Escherichia coli MTCC 40	21±0.4	22±1.6	-
Bacillus cereus MTCC 1272	16±0.9	28±1.7	-
Staphylococcus epidermidis MTCC 435	16±0.8	20±1.8	3.90
Staphylococcus aureus MTCC 740	22±1.4	30±0.6	3.90
**Fungi**		**(Nystatin)**	
Aspergillus flavus MTCC 2590	-	-	-
Bipolaris maydis	-	-	-
Alternaria alternata MTCC 1362	-	-	-
Fusarium moniliforme MTCC 6576	14±1.0	20±0.7	62.50

Table S2. α-glucosidase inhibition and IC_{50} values of pyrrolo[1,2-a]pyrazine-1,4-dione,hexahydro-3-(2-methylpropyl) (Commercial compound)

Concentration (µg/ml)	Inhibition % (Commercial compound)	IC_{50} (µg/ml) (Commercial compound	Inhibition % (Acarbose)	IC_{50} (µg/ml) (Acarbose)
6.25	14.11±2.16		30.21±0.33	
12.5	16.03±1.90		41.01±0.62	
25	23.90±0.64	117.80	61.74±1.51	17.91
50	38.12±1.10		70.02±1.12	
100	48.23±1.01		84.83±2.36	
200	61.22±0.13		88.63±2.33	

Table S3. α-amylase inhibition and IC_{50} values of pyrrolo[1,2-a]pyrazine-1,4-dione,hexahydro-3-(2-methylpropyl) (Commercial compound)

Concentration (µg/ml)	Inhibition % (Commercial compound)	IC_{50} (µg/ml) (Commercial compound)	Inhibition % (Acarbose)	IC_{50} (µg/ml) (Acarbose)
	**EA extract**		**Acarbose**	
6.25	5.30±0.91		16.43±1.18	
12.5	16.32±0.37		31.30±0.60	
25	20.14±1.18	164.24	57.04±2.01	21.07
50	29.47±2.06		79.01±0.61	
100	44.73±2.34		81.06±0.51	
200	51.97±0.93		91.41±1.29	
